# Neonatal Therapy Interventions Supporting Oral Feeding Skills in Preterm Infants: A Systematic Review

**DOI:** 10.1080/01942638.2025.2562931

**Published:** 2025-09-29

**Authors:** Kaela Vetter, Christian Fernandes, Tiana T. Nguyen

**Affiliations:** a Department of Occupational Therapy, Samuel Merritt University, Oakland, CA, USA; b Department of Rehabilitation, John Muir Health, Walnut Creek, CA, USA; c Department of Rehabilitation, Kaiser Permanente, Redwood City, CA, USA; d Department of Physical Therapy and Rehabilitation Sciences, University of California, San Francisco, San Francisco, CA, USA

**Keywords:** Feeding therapy, occupational therapy, oral motor development, physical therapy, speech-language pathology

## Abstract

**Aim:**

To synthesize the existing literature on neonatal therapy interventions and evaluate their impact on oral feeding outcomes among preterm infants in the neonatal intensive care unit.

**Methods:**

A systematic literature search was conducted in PubMed, Cumulative Index to Nursing and Allied Health Literature (CINAHL), Web of Science, and Physiotherapy Evidence Database (PEDro) databases for studies published from 2014 to 2023. Included studies were clinical trials evaluating neonatal therapy interventions delivered by neonatal therapists or caregivers trained by therapists, focusing on oral feeding outcomes for infants born at <37 week gestation. Primary outcomes were time to full oral feeding (FOF) and feeding quality. Secondary outcomes were weight gain and length of stay (LOS). Risk of bias was assessed using the Cochrane Risk of Bias 2 tool.

**Results:**

Fourteen studies were included, identifying three categories of neonatal therapy interventions: oral motor stimulation, swallowing exercises, and sensory-based interventions. Most interventions began between 27 and 33 week postmenstrual age. Interventions generally resulted in improved oral feeding outcomes, notably faster achievement of FOF and improved feeding quality. Results for LOS and weight gain varied. Multidomain interventions demonstrated superior outcomes compared to single-domain approaches.

**Conclusion:**

Neonatal therapy interventions can start early and improve oral feeding outcomes in preterm infants, particularly when using multi-domain approaches.

## Introduction

According to the World Health Organization (2023), an estimated 13.4 million infants worldwide were born preterm (<37 week gestation) in 2020. Preterm infants are at higher risk for neurodevelopmental impairments and feeding difficulties compared to term infants, and these risks are magnified at lower gestational ages (Centers for Disease Control and Prevention, 2024). Prematurity impacts the development of non-nutritive sucking (NNS) and suck–swallow–breathe coordination due to immature physiological structures, neuromotor functions, neurobehavioral disorganization, respiratory demands (Lau, 2016), and negative orofacial stimuli from necessary medical procedures and routine care (Edwards et al., 2019; Pickier et al., 2013). Up to 80% of preterm infants experience feeding difficulties (Pados et al., 2021; Pineda et al., 2020), leading to prolonged neonatal intensive care unit (NICU) stays. Approximately, 37% of preterm infants remain hospitalized at 36 weeks due to the inability to attain full oral feeding (FOF; Bakewell-Sachs et al., 2009; Edwards et al., 2019). Beyond immediate hospitalization impacts, oral feeding challenges also have implications for long-term neurodevelopmental outcomes and family stress (Jadcherla et al., 2017; Warren et al., 2019).

Given the high neuroplasticity in preterm infants, timely rehabilitation interventions targeting feeding skills are critical for optimizing developmental outcomes (Butera et al., 2023; Liu et al., 2021; Nguyen et al., 2025; Pickier et al., 2013; Ross et al., 2017; Vasung et al., 2019). Neonatal therapists are licensed occupational therapists, physical therapists, and speech-language pathologists who have specialized training in providing rehabilitation interventions to preterm and medically complex infants in the NICU. These neonatal therapy interventions include sensory and motor-based feeding interventions, caregiver education, environmental adjustments, and neurobehavioral supports aimed at promoting oral feeding skills (National Association of Neonatal Therapists, 2022).

Although neonatal therapy can start early at lower postmenstrual ages (PMAs), its utilization varies across hospitals due to staffing availability and clinician expertise (Butera et al., 2023; Nguyen et al., 2025; Ross et al., 2017). Furthermore, there are limited guidelines and evidence on the optimal timing, frequency, and duration of these interventions, specifically for feeding outcomes. To date, only one study has broadly evaluated neonatal therapy interventions on cognitive, motor, and behavioral outcomes (Khurana et al., 2020), but none have exclusively focused on oral feeding outcomes. This systematic review aims to address this gap by evaluating the impact of neonatal therapy interventions specifically on oral feeding outcomes in preterm infants. Additionally, the review will synthesize information on the timing, duration, and frequency of interventions to inform evidence-based clinical practice for neonatal therapists in NICU settings.

## Methods

### Study Design

This systematic review was developed according to the Preferred Reporting Items for Systematic Reviews and Meta Analyses guidelines (Page et al., 2021). The research question was developed using the Population, Intervention, Comparison, Outcome framework (Schardt et al., 2007). Peer-reviewed clinical trials published in English between 1 January 2014 and 31 December 2023 were included to capture recent advancements in neonatal therapy. The final database search was conducted on 31 January 2024.

The research question was: In hospitalized preterm infants (<37 week gestation) in the NICU, do habilitative neonatal therapy interventions delivered by neonatal therapists (occupational therapists, physical therapists, or speech-language pathologists) improve oral feeding outcomes compared to routine care or sham interventions? The primary outcomes were time to FOF, age at FOF, use of feeding tube at discharge, and feeding quality (including feeding efficiency, spillage, volume, feeding readiness, strength of suction, state regulation, and physiological stability during feeding). The secondary outcomes were weight gain and length of stay (LOS).

The study population included hospitalized preterm infants born at <37 week gestation receiving care in the NICU. Eligible interventions were habilitative neonatal therapy interventions, defined as therapeutic activities aimed at skill development and provided directly by neonatal therapists or caregivers trained by neonatal therapists. Interventions such as feeding modifications and compensatory strategies aimed at accommodating feeding difficulties without promoting skill development were excluded. Both breast- and bottle-feeding outcomes were considered. Studies focusing exclusively on enteral feeding outcomes were excluded.

### Search Strategy

We conducted a systematic search of PubMed, Cumulative Index to Nursing and Allied Health Literature (CINAHL), Web of Science, and Physiotherapy Evidence Database (PEDro) on 31 January 2024. Search concepts included premature or preterm infants; neonatal therapy, rehabilitation, or feeding interventions; and clinical trials and randomized controlled trials. Searches were limited to studies published in English from 1 January 2014 to 31 December 2023, focusing on oral feeding outcomes in preterm infants (<37 week gestation) in the NICU. A detailed description of the database search strategy is provided in Supplementary Table S1. Citations were exported into Mendeley. Duplicates were automatically identified and removed, then manually verified.

### Study Selection

Two reviewers independently screened titles and abstracts against inclusion criteria using a standardized spreadsheet. Full texts were retrieved and reviewed when eligibility could not be clearly determined from titles and abstracts. Disagreements were resolved through discussion with a third reviewer.

### Data Extraction/Analysis

Two reviewers independently extracted data using a structured matrix. Extracted data included author(s), article title, journal, publication year, study purpose, participant characteristics (birth gestational age and weight), country, study design, outcome measures, control conditions, intervention specifics (type, provider, timing, frequency, duration), PMA at the initiation of intervention, outcomes (primary and secondary), and study limitations. Studies were grouped by the primary intervention type. Discrepancies in data extraction were resolved by consensus among the reviewers. Cross-verification of the final reference list was performed to ensure data accuracy. For the purposes of this review, we defined statistical significance as *p* < .05.

### Quality Assessment and Risk of Bias

Study quality and risk of bias were assessed using the Cochrane Risk of Bias 2.0 (RoB 2) tool for randomized trials (Higgins et al., 2024). Key domains assessed included randomization process, deviations from intended interventions, missing outcome data, measurement of outcomes, and selection of reported results. Two reviewers independently assigned each study a risk of bias rating (“low,” “some concerns,” or “high”). Any disagreements were reviewed and resolved through discussion. All studies rated as “low” or “some concerns” were included in this review.

## Results

**Figure 1.**
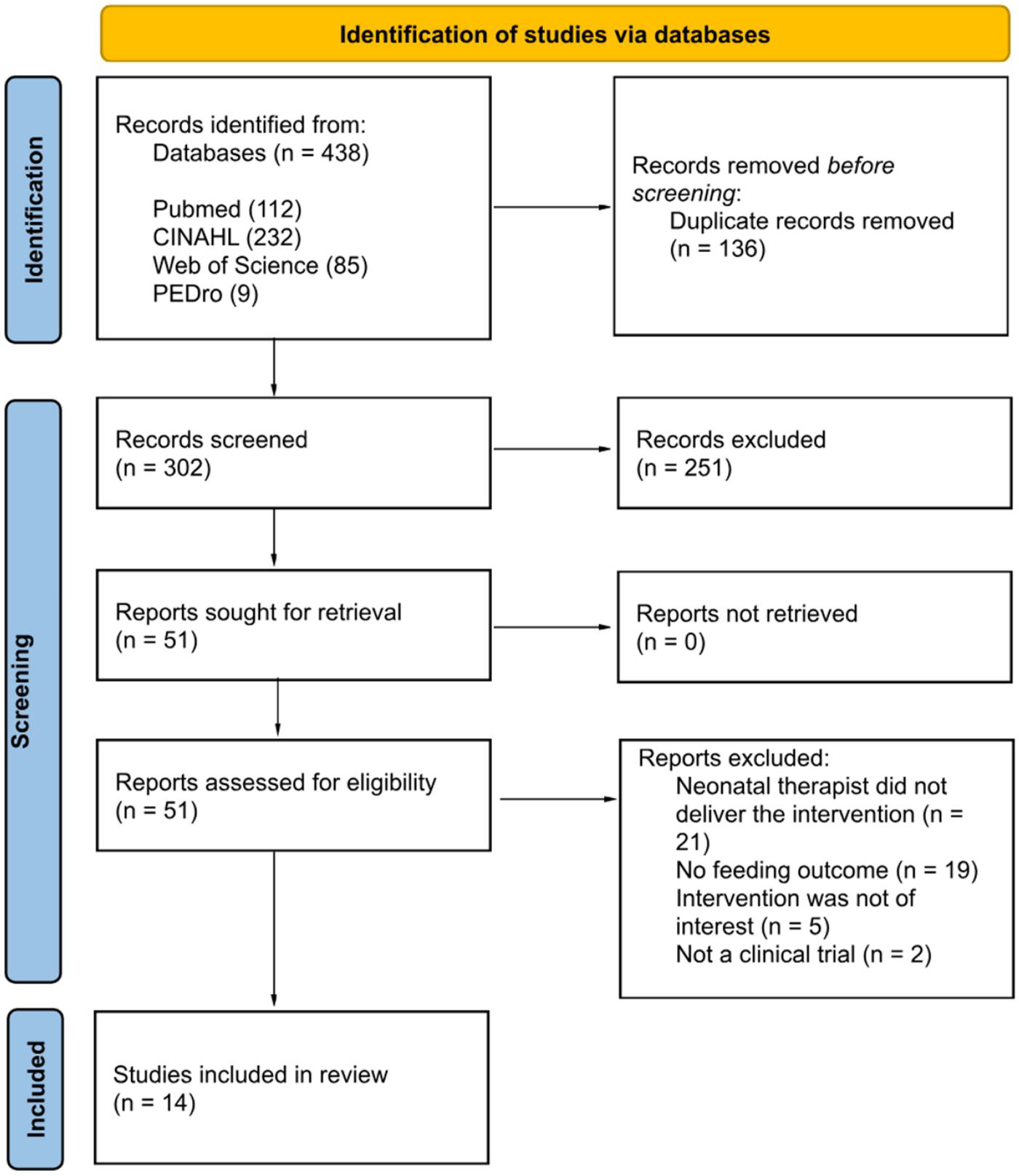
PRISMA Flow diagram for articles included in systematic review. *Note*. Adapted from Page et al. (2021).

A total of 438 results were retrieved. Of these, 51 studies underwent full-text review, and 14 met the inclusion criteria (see Figure 1). Risk of bias assessments using the RoB 2 tool indicated that nine studies had a “low” risk of bias, five studies had “some concerns,” and none had a “high” risk of bias (see Table 1). Therefore, all 14 articles were included in this systematic review.

The included studies were conducted across diverse geographic locations: Iran (Alidad et al., 2021; Ghomi et al., 2019; Ostadi et al., 2021; Shokri et al., 2023), India (John et al., 2018; Thakkar et al., 2018), Spain (Aguilar-Rodríguez et al., 2020; Hernández Gutiérrez et al., 2022), the United States (Song et al., 2019), South Korea (Heo et al., 2022), Denmark (Skaaning et al., 2020), Brazil (da Rosa Pereira et al., 2020), Italy (Majoli et al., 2023), and the United Kingdom (Harding et al., 2014). Sample sizes ranged from 30 to 210 infants, with birth gestational ages spanning 25–36 weeks. Interventions that neonatal therapists provide that address oral feeding include: (1) oral motor stimulation (OMS); (2) swallowing exercises; and (3) sensory-based interventions (see Table 2). Findings are detailed in Supplementary Table S2.

### Oral Motor Stimulation

OMS includes massage, strokes, and tactile stimulation of the intra-oral, peri-oral, and orofacial regions to improve oral motor strength and coordination for sucking (Fucile et al., 2002, 2005; Greene et al., 2023; Lessen, 2011). Developing effective sucking skills is essential as it enables infants to extract milk during oral feeding efficiently. Key OMS techniques evaluated included Premature Infant Oral Motor Intervention (PIOMI), the Fucile protocol, and NNS exercises.

### Premature Infant Oral Motor Intervention

PIOMI is an oral motor intervention for preterm infants that can start as young as 29 week gestation (Lessen, 2011). It is a standardized protocol that involves five minutes of stretching, rolling, pressing, and/or stroking of lips, gums, cheeks, tongue, and palate, followed by elicitation of NNS with a finger or pacifier. PIOMI is typically administered daily for seven consecutive days leading up to initiating oral feeding attempts (Lessen, 2011).

Three studies evaluated the effectiveness of PIOMI compared to standard care (Ghomi et al., 2019; Skaaning et al., 2020; Thakkar et al., 2018), while one study compared PIOMI administered by caregivers trained by neonatal therapists to direct implementation by neonatal therapists (Majoli et al., 2023). Modifications were made to the original PIOMI protocol including the timing of the initiation and the duration of the intervention (Ghomi et al., 2019; Skaaning et al., 2020; Thakkar et al., 2018). PIOMI was initiated as early as 29 week PMA and was initiated before or after the start of oral feeding. PIOMI was performed 1–2 times daily for at least seven days or until reaching FOF (Ghomi et al., 2019; Skaaning et al., 2020; Thakkar et al., 2018).

Infants who received PIOMI achieved FOF approximately 5–6 days earlier, were discharged 2–9 days earlier, and demonstrated improved feeding efficiency compared to controls (Ghomi et al., 2019; Thakkar et al., 2018). However, Skaaning et al. (2020) found no significant impact on suck strength, and results related to weight gain were inconsistent across studies (Ghomi et al., 2019; Thakkar et al., 2018). Additionally, PIOMI administered by caregivers trained by neonatal therapists showed outcomes comparable to interventions provided directly by neonatal therapists (Majoli et al., 2023).

### Fucile Protocol

The Fucile protocol consists of 12 min of stroking of the cheeks, lips, gums, and tongue, followed by three minutes of NNS on a pacifier. It is typically administered during the 10 days leading up to the initiation of oral feeding (Fucile et al., 2002, 2005). Two studies compared the effectiveness of the Fucile protocol with routine care (Aguilar-Rodríguez et al., 2020; da Rosa Pereira et al., 2020), while a third study compared outcomes when the protocol was implemented by neonatal therapists versus caregivers trained by neonatal therapists (John et al., 2018). Modifications to the original protocol included alterations to the sequence of oral stimulation maneuvers and adjustments to the intervention duration (Aguilar-Rodríguez et al., 2020; John et al., 2018). The Fucile protocol was initiated between 30 and 31 week PMA or when infants achieved physiological stability. The intervention occurred once daily for at least 10 days or continued until discharge, starting either before or after oral feeding attempts had begun (Aguilar-Rodríguez et al., 2020; da Rosa Pereira et al., 2020; John et al., 2018).

Two studies showed that infants who received the Fucile protocol achieved FOF 4–6 days faster and had better feeding efficiency compared to routine care (Aguilar-Rodríguez et al., 2020; da Rosa Pereira et al., 2020). However, findings related to LOS and weight gain were mixed across these studies (Aguilar-Rodríguez et al., 2020; da Rosa Pereira et al., 2020). Additionally, no differences in outcomes were identified between implementations conducted by neonatal therapists versus those by caregivers trained by neonatal therapists (John et al., 2018).

### Non-Nutritive Sucking

Oral feeding requires the maturation of a complex set of skills allowing the infant to coordinate the suck–swallow–breathe cycle (Barlow, 2009). NNS involves rhythmic sucking on a pacifier, finger, or empty breast without the ingestion of breastmilk or formula, allowing the infant to practice and refine sucking movements independently of swallowing and respiratory demands (Barlow, 2009; Medeiros et al., 2011).

The reviewed studies implemented NNS interventions using a variety of methods, including pulsatile pacifiers (Song et al., 2019), non-pulsatile pacifiers (Harding et al., 2014), or therapist’s gloved finger (Ostadi et al., 2021). Song et al. (2019) specifically used the NTrainer oral training regimen. NNS interventions were typically initiated between 27 and 32 week PMA or when infants were showing signs of oral readiness. The interventions were conducted 2–4 times daily, lasting 5–20 min per session, and were continued for at least two weeks or until the infant achieved FOF (Harding et al., 2014; Song et al., 2019).

Overall, NNS interventions generally promoted faster transition times to FOF and improved feeding readiness. However, findings regarding LOS were mixed. One study found no improvement in time to FOF but reported a reduced LOS (Harding et al., 2014), whereas other studies demonstrated quicker transitions to FOF without significant effects on LOS (Ostadi et al., 2021; Song et al., 2019). Additionally, pulsatile pacifiers showed superior outcomes compared to non-pulsatile pacifiers (Song et al., 2019).

### Swallowing Exercise

Effective swallowing coordination within the suck–swallow–breathe cycle is critical for airway protection, respiratory stability, and safe and efficient feeding (Arvedson, 2006; Barlow, 2009). Swallowing exercises involve the repeated practice of coordinated swallowing movements using small boluses of breastmilk or formula to promote the development of oral feeding skills (Lau & Smith, 2012). Lau and Smith (2012) developed a structured swallowing exercise protocol in which a 0.05–0.2 mL milk bolus is delivered by syringe to the medial-posterior aspect of the infant’s tongue every 30 s, eliciting repetitive swallows over a 15-minute period.

Two studies evaluated the effectiveness of swallowing exercises on oral feeding outcomes (Heo et al., 2022; Ostadi et al., 2021). Swallowing exercises were implemented either alone or combined with OMS. Interventions began between 27 and 30 week PMA and were performed daily for 15 min over a 10-day period (Ostadi et al., 2021) or until the infant reached FOF (Heo et al., 2022).

Overall, infants receiving swallowing exercises, either alone or combined with OMS, had greater improvements in several feeding outcomes compared to routine care, with greater improvements generally observed when swallowing exercises were combined with OMS (Heo et al., 2022; Ostadi et al., 2021). In Heo et al. (2022), infants receiving swallowing exercises combined with OMS achieved FOF about six days earlier than controls and demonstrated higher feeding proficiency at the initiation of oral feeding (mean difference = 8%). No significant differences were found in LOS or weight gain. In Ostadi et al. (2021), swallowing exercises combined with NNS increased the likelihood of discharge without tube feeding (78.6% vs. 30.8%) and improved feeding readiness, though differences in time to FOF and PMA at discharge did not reach significance.

### Sensory-Based Interventions

Sensory-based interventions were administered in combination with OMS or during oral feeding. These interventions included tactile (Alidad et al., 2021; Hernández Gutiérrez et al., 2022), kinesthetic (Hernández Gutiérrez et al., 2022), and auditory stimulation (Shokri et al., 2023) starting between 30 and 33 week PMA. Sensory-based interventions combined with OMS were delivered every other day or up to twice daily for durations ranging from 10 to 14 days (Alidad et al., 2021; Hernández Gutiérrez et al., 2022; Shokri et al., 2023).

Overall, infants receiving combined sensory-based and OMS interventions achieved FOF 4–9 days faster, demonstrated improved feeding readiness, and consumed higher milk volumes compared to those receiving OMS alone (Alidad et al., 2021; Hernández Gutiérrez et al., 2022; Shokri et al., 2023). However, no significant differences were observed for LOS or weight gain outcomes (Alidad et al., 2021; Hernández Gutiérrez et al., 2022; Shokri et al., 2023).

## Discussion

This systematic review evaluated the impact of neonatal therapy interventions on oral feeding outcomes in preterm infants in the NICU. We also described the timing of initiation, duration, and frequency of these interventions. Findings suggest that neonatal therapy can improve oral feeding outcomes including time to FOF, feeding readiness, and feeding efficiency in this high-risk population. However, impacts on weight gain and LOS were variable.

Our review highlights that neonatal therapy interventions can begin as early as 27 week PMA, with most studies initiating interventions between 30 and 33 week PMA. This is consistent with existing research that neonatal therapy can begin at young PMAs (Butera et al., 2023; Nguyen et al., 2025; Ross et al., 2017). Given the high neuroplasticity of preterm infants and the increased risk of feeding delays due to low muscle tone, medical complications, and immature body systems, early intervention is critical to support the development of oral motor and swallowing skills.

Neonatal therapists deliver a range of interventions including OMS, swallowing exercises, and sensory-based approaches to facilitate oral feeding skill development. A few included studies compared combined interventions with their single components (e.g. sensory-based stimulation plus OMS vs. OMS alone; swallowing exercises plus OMS vs. swallowing exercises alone). Across these studies, the pattern of results suggests that adding complementary modalities enhances oral feeding readiness, progression, and efficiency. This observation aligns with the understanding that successful oral feeding requires coordination across various body systems (Arvedson, 2006; Barlow, 2009). Although none of the included studies directly compared multimodal with unidimensional interventions across all domains, studies that tested combined versus single components generally reported better outcomes with combined approaches. Taken together, these findings suggest that multimodal interventions could be more effective. Future trials are needed to directly compare single- versus multi-component interventions to determine whether multimodal strategies confer greater benefits.

The reviewed studies spanned diverse geographic regions with varying neonatal therapy practices, professional disciplines (occupational therapy, physical therapy, speech-language pathology), and resource availability. Variations in service delivery highlight the importance of caregiver training as a key component of neonatal therapy practice to enhance intervention consistency and carryover (NANT, 2022; Sturdivant, 2013). Previous research demonstrates that caregiver education significantly improves caregiver confidence and infant feeding outcomes (Cooper et al., 2007; Craig et al., 2015; Giannì et al., 2016; Springer et al., 2023). However, specialized expertise provided by neonatal therapists remains critical for individualized care planning, especially given the complex developmental trajectories and medical conditions of preterm infants (NANT, 2022; Sturdivant, 2013).

Most included studies reported positive effects of neonatal therapy interventions, suggesting strong support for their use in clinical settings. Two studies did not find significant differences compared to control groups. Specifically, Skaaning et al. (2020) limited outcome measures to suction strength and exclusive breastfeeding rates without examining broader outcomes like FOF timing, potentially limiting the intervention’s measurable impact. Harding et al. (2014) reported no significant benefit of NNS intervention, contrasting with extensive existing evidence demonstrating positive effects of NNS (Arvedson et al., 2010; Foster et al., 2016; Zhang et al., 2014). There are several limitations in Harding et al. (2014) leading to some concerns with bias, including lack of documented baseline comparisons between groups, variability in personnel administering interventions, and unclear reporting of intervention frequency.

### Limitations

Several limitations should be considered when interpreting the findings of this systematic review. First, because we excluded studies not available in English, we may have missed relevant evidence published in other languages, which could limit the comprehensiveness and generalizability of our findings. Second, substantial variability in intervention type, frequency, duration, and protocols prevented us from conducting a meta-analysis. Such variability also makes it challenging to isolate specific components contributing to intervention effectiveness. Additionally, the inability to blind therapists and caregivers delivering or receiving habilitative interventions inherently increases the risk of bias. This review focused exclusively on habilitative interventions and did not evaluate compensatory feeding strategies. Moreover, the studies reviewed primarily addressed short-term feeding outcomes during NICU hospitalization, highlighting the need for longitudinal research to examine long-term impacts after NICU discharge.

Another important limitation is that included studies explicitly excluded preterm infants with severe comorbidities such as grade III/IV intraventricular hemorrhage, bronchopulmonary dysplasia, necrotizing enterocolitis, genetic or chromosomal conditions, seizures, congenital malformations, and sepsis. While excluding infants with these comorbidities minimizes confounding factors, it also significantly restricts the generalizability. These medical complications are prevalent among preterm populations (Alsaied et al., 2020; Jensen & Schmidt, 2014; Piccolo et al., 2022; Pisani & Spagnoli, 2019) and significantly impact oral feeding development and outcomes (Barlow, 2009; Edwards et al., 2019). Future research is critically needed to examine neonatal therapy interventions in medically complex infants, for whom feeding outcomes may be particularly vulnerable.

### Clinical Implications

The findings of this review highlight several important clinical implications:

1. Early initiation of habilitative interventions should be prioritized to optimize feeding outcomes among preterm infants.
2. Neonatal therapists should systematically incorporate oral motor, swallowing, and sensory-based habilitation strategies into standard NICU care, especially for infants at high risk for feeding difficulties.
3. Active involvement of caregivers in therapeutic interventions is essential, as empowering and educating caregivers may enhance skill acquisition and lead to improved infant feeding outcomes.
4. A multimodal therapeutic approach that includes OMS, sensory-based interventions, and swallowing exercises may offer greater benefits than isolated interventions.
5. Neonatal therapists should create individualized intervention plans to address each infant’s unique medical, developmental, and sensory-motor profile.

To our knowledge, this is the first systematic review specifically examining interventions implemented by neonatal therapists that impact oral feeding outcomes in preterm infants in the NICU. The findings add to the body of evidence demonstrating that neonatal therapy interventions, particularly those involving multiple domains and caregiver involvement, support oral feeding outcomes. This review emphasizes the feasibility and importance of initiating interventions at young PMAs and highlights the value of early, proactive involvement of neonatal occupational therapists, physical therapists, and speech-language pathologists. Additional research is necessary to identify optimal intervention frequency, duration, and effectiveness for preterm infants with significant medical complexities and severe comorbidities.

## Conclusion

Early neonatal therapy interventions, including OMS, NNS, swallowing exercises, sensory-based interventions, and caregiver-implemented strategies, significantly improve oral feeding outcomes in preterm infants. Interventions that combine multiple neonatal practice domains appear to yield the most favorable results. Further studies are necessary to establish optimal intervention frequencies, durations, and specific strategies best suited for infants with severe medical complexities and comorbidities.

## Supplementary Material

Supp 1

Supp 2

Supplemental data for this article can be accessed online at https://doi.org/10.1080/01942638.2025.2562931.

## Figures and Tables

**Table 1.** Risk of bias assessment.

Reference	Bias from the randomization process	Deviations from intended intervention (effect of assignment to intervention)	Deviations from intended intervention (effect of adhering to intervention)	Bias from missing outcome data	Bias in measurement of the outcome	Bias in selection of reported result	Overall risk of bias

Aguilar-Rodríguez et al. (2020)	Low risk	Low risk	Low risk	Low risk	Low risk	Low risk	Low risk
Alidad et al. (2021)	Low risk	Low risk	Low risk	Low risk	Low risk	Low risk	Low risk
da Rosa Pereira et al. (2020)	Low risk	Low risk	Low risk	Low risk	Low risk	Low risk	Low risk
Ghomi et al. (2019)	Some concern	Low risk	Low risk	Low risk	Low risk	Low risk	Some concern
Harding et al. (2014)	Some concern	Some concern	Some concern	Low risk	Low risk	Low risk	Some concern
Heo et al. (2022)	Some concern	Low risk	Low risk	Low risk	Low risk	Low risk	Some concern
Hernández Gutiérrez et al. (2022)	Some concern	Low risk	Low risk	Low risk	Low risk	Low risk	Some concern
John et al. (2018)	Low risk	Low risk	Low risk	Low risk	Low risk	Low risk	Low risk
Majoli et al. (2023)	Low risk	Low risk	Low risk	Low risk	Low risk	Low risk	Low risk
Ostadi et al. (2021)	Some concern	Low risk	Low risk	Low risk	Low risk	Low risk	Some concern
Shokri et al. (2023)	Low risk	Low risk	Low risk	Low risk	Low risk	Low risk	Low risk
Skaaning et al. (2020)	Low risk	Low risk	Low risk	Low risk	Low risk	Low risk	Low risk
Song et al. (2019)	Low risk	Low risk	Low risk	Low risk	Low risk	Low risk	Low risk
Thakkar et al. (2018)	Low risk	Low risk	Low risk	Low risk	Low risk	Low risk	Low risk

**Table 2.** Summary of participant and intervention characteristics.

Intervention	Reference	Participant inclusion criteria	Initiation of therapy	Intervention description

Oral motor stimulation	Aguilar-Rodríguez et al. (2020), Spain	GA of 25–30 weeks, requiring gavage feeding, with hemodynamic and cardiorespiratory clinical stability, and without any associated severe pathology. Infants with congenital anomalies affecting feeding, abnormalities that altered digestive function, babies suffering severe sepsis or culture of positive meningitis, chronic medical complications or difficulty in identifying an accurate GA were excluded.	30–34 week PMA	EG: Fucile protocol implemented for 10 min over a two week period (20 sessions in total) prior to oral feeding attempts. CG: routine care
	da Rosa Pereira et al. (2020), Brazil	GA of 26–32 weeks. Excluded congenital malformations, IVH grade III/IV, BPD, and NEC.	31 week PMA and when clinically stable	EG: Fucile protocol implemented for 15 min daily for 10 consecutive days prior to oral feeding attempts. CG: sham intervention
	Ghomi et al. (2019), Iran	GA of 26–29 weeks with Apgar score of 6 at 5 min after birth. Excluded congenital disorders, chromosomal anomalies, BPD, IVH III/IV, NEC, seizures, asphyxia, neonatal jaundice necessitating exchange transfusion, and proven sepsis with positive blood culture, or prescribed with gavage feeding before week 29.	29 week PMA, after the pediatrician allowed for gavage feeding, and the infant was physiologically stable before, during, and after oral stimulations.	EG: PIOMI intervention 1×/day for 10 days CG: routine care
	Harding et al. (2014), United Kingdom	GA of 26–35 weeks without congenital disorders, IVH III/IV, severe respiratory problems, or NEC.	Initiated when infants started to show signs of “oral readiness”	NNS on a pacifier implemented for a minimum of 5min and a minimum of three times per day Group I: NNS pre-tube feeding Group II: NNS on onset of tube feeding Group III (CG): routine care
	John et al. (2018), India	GA of ≤32 weeks without congenital and/or chromosomal diseases.	Initiated prior to oral feeding attempts when infants were physiologically stable, tolerating NG feeds, and reached postnatal weight of 1250 g	EG: caregiver trained by a neonatal therapist implemented the Fucile protocol 1×/day for 30 min before tube or oral feeding until discharge. CG: neonatal therapist implemented the Fucile protocol 1×/day for 30 min before tube or oral feeding until discharge.
	Majoli et al. (2023), Italy	GA of ≤32 weeks.	31–32 week PMA prior to oral feeding attempts. Infants had to be clinically stable and require FiO_2_ ≤ 0.25, or high-flow nasal cannula ≤3L/min, and tolerate tube feeding of at least 30 mL/kg/day.	EG: caregiver trained by a neonatal therapist implemented PIOMI 1×/day for 7 consecutive days CG: neonatal therapist implemented PIOMI 1×/day for 7 consecutive days
	Skaaning et al. (2020), Denmark	GA of <37 weeks, breastfeeding intended, no congenital malformations, BPD, or other diseases that complicate breastfeeding.	≥32 week PMA	EG: caregiver trained by a neonatal therapist implemented modified PIOMI 2×/day for 14 days and continued for a longer period if the stimulation seemed “appropriate” CG: routine care
	Song et al. (2019), USA	GA of 26 0/7 to 30 6/7 weeks without chromosomal or congenital anomalies, meningitis, seizures, NEC ≥ Bell stage 2, vocal cord paralysis. Infants who were already taking feeds orally were excluded.	30–32 week PMA and once infant was medically stable	Intervention period lasted for two weeks or when an infant reached FOF, whichever came first. Infants in both groups received the intervention up to four times per day EG: 20 min NTrainer oral training regimen which turns the pacifier into a pulsating pacifier CG: oral suck training with the same pacifier without the pulsatile effect
	Thakkar et al. (2018), India	GA of 30–34 weeks, hemodynamically stable, and receiving at least 100 mL/kg of mother’s milk as gavage feeds. Infants with medical complications such as IVH III/IV, severe perinatal asphyxia, severe sepsis, congenital disease, or malformation, and those on formula feeds were excluded.	Initiated at first oral feed (~33 week PMA)	EG: PIOMI implemented 2×/day 15 min prior to oral feeding. Implemented until the infant achieved FOF CG: routine care
Swallowing exercise	Heo et al. (2022), South Korea	GA of <32 weeks who achieved full tube feeding and weaned from NCPAP before 33 week PMA. Infants with major congenital anomalies, gastrointestinal complications, or chronic medical conditions such as IVH III/IV, PVL, and surgical NEC were excluded.	32 weeks PMA	Implemented until the infant reached FOF. Group I (CG): sham intervention 15 min 2×/day, Group II: sham intervention 15 min 1×/day + DST 15 min 1×/day, Group III: Fucile protocol 15 min 1×/day + DST 15 min 1×/day
	Ostadi et al. (2021), Iran	Infants with 27–30 PMA without congenital anomalies or craniofacial malformations who received all feedings by tube, did not have ventilator or high-flow oxygen dependency, and had physiological stability. Infants with IVH, NEC, and BPD were excluded.	27–30 week PMA	Implemented for 10 days. Group I: NNS for 15 min 2×/day. Group II: SE for 15 min 1×/day and NNS for 15 min 1×/day. Group III (CG): routine care
Sensory-based	Alidad et al. (2021), Iran	GA of 30–36 weeks with presence of feeding problems and feeding via NG tube.	30 week PMA after pediatrician allowed for gavage feeding and the infant was medically stable	Implemented for 14 consecutive days 30 min before scheduled tube feeding. EG: NNS for 5 min 7–8×/day, the Fucile protocol for 12 min 1×/day and tactile support (chin and jaw support) during oral feeding 10 min 2×/day. CG: NNS for 5 min 7–8×/day
	Hernández Gutiérrez et al. (2022), Spain	GA of 27–32 weeks, birth weight ≥900 g, weight adequate for GA, NG/OG tube feeding, hemodynamic and clinical stability, and absence of nutritive sucking. Infants with congenital orofacial anomalies, IVH III/IV, post hemorrhagic hydrocephalus, PVL, severe systemic disease (such as sepsis or NEC), major surgery and invasive mechanical ventilation with endotracheal intubation during the intervention were excluded.	32–33 week PMA when in quiet alert state	Intervention implemented for 15 min 1×/day for 10 days for both groups EG: alternated days for Fucile protocol and tactile + kinesthetic stimulation. Tactile stimulation involved five minutes of infant massage and facilitation into physiological flexion position. Kinesthetic stimulation involved gentle passive range of motion of bilateral upper and lower extremities for 10 min. CG: Fucile protocol
	Shokri et al. (2023), Iran	GA of 26–30 weeks, physiological respiratory and cardiovascular stability vital signs, and lack of stress symptoms when presenting stimuli. Infants with intubation or assisted ventilation like CPAP, Apgar score less than seven at birth and 5 min after birth, congenital anomalies and chronic medical problems such as asphyxia and seizures, BPD, IVH, NEC, or hearing loss were excluded.	30 week PMA when physiologically stable and had initiated gavage feeding	Intervention implemented 1×/day for 10 days. EG: 5 min PIOMI followed by 10 min of music therapy, which consisted of playing 10 min of Mozart music at 45 ± 5dB with the speaker positioned 30 cm from the infant’s ear. CG: 5 min PIOMI

GA: gestational age; PMA: postmenstrual age; EG: experimental group; CG: control group; IVH: intraventricular hemorrhage; BPD: bronchopulmonary dysplasia; NEC: necrotizing enterocolitis; PIOMI: Premature Infant Oral Motor Intervention; NNS: nonnutritive sucking; NG: nasogastric; FOF: full oral feeding; NCPAP: nasal continuous positive airway pressure; PVL: periventricular leukomalacia; DST: direct swallowing training; SE: swallowing exercise; OG: orogastric; CPAP: continuous positive airway pressure.
