# Supplementary material for: Neonatal Therapy Interventions Supporting Oral Feeding Skills in Preterm Infants: A Systematic Review
Source: Phys Occup Ther Pediatr. Author manuscript; Available in PMC 2025 Oct 31. (PMC12573778; doi:10.1080/01942638.2025.2562931)
Supplement: Supp 1 [file NIHMS2114797-supplement-Supp_1.docx]

**Table 1.** Database Search String and Filters

| Database | Search string | Filters |
| --- | --- | --- |
| PubMed | (("Infant, Premature"[Mesh] OR "Infant, Low Birth Weight"[Mesh] OR prematur*[tiab] OR preterm[tiab] OR premie*[tiab] OR preemie*[tiab] OR "low birth weight"[tiab] OR neonat*[tiab] OR "NICU"[tiab]) AND  ("Feeding Behavior"[Mesh] OR "Feeding Methods"[Mesh] OR "Eating"[Mesh] OR "Sucking Behavior"[Mesh] OR "Deglutition"[Mesh] OR feed*[tiab] OR eat*[tiab] OR "food intake"[tiab] OR suck*[tiab] OR swallow*[tiab] OR "oral feeding"[tiab] OR "feeding efficiency"[tiab] OR "feeding readiness"[tiab]) AND  ("Physical Therapy Modalities"[Mesh] OR "Rehabilitation"[Mesh] OR "Speech Therapy"[Mesh] OR "Occupational Therapy"[Mesh] OR "Massage"[Mesh] OR "Physical Stimulation"[Mesh] OR "Cues"[Mesh] OR "Pacifiers"[Mesh] OR "oral motor"[tiab] OR "oral stimulation"[tiab] OR "swallowing exercise"[tiab] OR "non-nutritive sucking"[tiab] OR "oral support"[tiab] OR piomi[tiab] OR ntrainer*[tiab] OR nfant*[tiab] OR massag*[tiab] OR pacifier*[tiab] OR "caregiver training"[tiab] OR "developmental care"[tiab]) | Publication date from 2014/01/01 to 2023/12/31; English; Humans; Clinical Trial, Randomized Controlled Trial |
| CINAHL | ((MH "Infant, Premature" OR MH "Low Birth Weight Infant" OR TX prematur* OR TX preterm OR TX premie* OR TX preemie* OR TX "low birth weight" OR TX neonat* OR TX NICU) AND (MH "Feeding Behavior" OR MH "Feeding Methods" OR MH "Eating" OR MH "Sucking Behavior" OR MH "Deglutition" OR TX feed* OR TX eat* OR TX "food intake" OR TX suck* OR TX swallow* OR TX "oral feeding" OR TX "feeding efficiency" OR TX "feeding readiness") AND (MH "Physical Therapy" OR MH "Rehabilitation" OR MH "Speech Therapy" OR MH "Occupational Therapy" OR MH "Massage" OR MH "Stimulation, Physical" OR MH "Sensory Stimulation" OR MH "Cues" OR MH "Pacifiers" OR TX "oral motor" OR TX "oral stimulation" OR TX "swallowing exercise" OR TX "non-nutritive sucking" OR TX "oral support" OR TX piomi OR TX ntrainer* OR TX nfant* OR TX massag* OR TX pacifier* OR TX "caregiver training" OR TX "developmental care")) | Language: English; Publication Type: Clinical Trial, Randomized Controlled Trial (RCT); Publication Years: 2014 to 2023 |
| Web of Science | TS=(prematur* OR preterm OR "low birth weight" OR neonat* OR NICU)  AND TS=("oral feeding" OR feed* OR suck* OR swallow* OR "feeding readiness" OR "feeding efficiency" OR "food intake" OR deglutition OR "feeding behavior")  AND TS=("oral motor" OR "oral stimulation" OR "swallowing exercise" OR "non-nutritive sucking" OR "feeding intervention" OR "feeding therapy" OR "developmental care" OR "caregiver training" OR "sensory stimulation" OR "neonatal therapy" OR "speech therapy" OR "occupational therapy" OR "physical therapy" OR massage OR stimulation OR pacifier* OR piomi OR ntrainer* OR nfant*) AND TS=("randomized controlled trial" OR "randomised controlled trial" OR "RCT" OR "clinical trial" OR "random allocation") | Timespan: 2014 to 2023; Document type: Article; Language: English; Research Areas: Pediatrics, Rehabilitation, Nursing |
| PEDro | Simple search term: preterm infant feeding intervention | Publication date from 2014 to 2023 |
