# Supplementary material for: Neonatal Therapy Interventions Supporting Oral Feeding Skills in Preterm Infants: A Systematic Review
Source: Phys Occup Ther Pediatr. Author manuscript; Available in PMC 2025 Oct 31. (PMC12573778; doi:10.1080/01942638.2025.2562931)
Supplement: Supp 2 [file NIHMS2114797-supplement-Supp_2.docx]

**Table S2.** Summary of Outcomes and Results

| **Intervention** | **Reference** | **Outcome Measure** | **Results** |
| --- | --- | --- | --- |
| Oral Motor Stimulation | Aguilar-Rodríguez et al. (2020), Spain | Time to FOF from initiation of intervention (days) | Median  EG: 36.0  CG: 42.5  *p*=0.013 |
|  |  | Oral intake (days to 30% oral intake in the first 5 minutes and 100% oral intake) | Mean ± SD  *Days to 30%*  EG: 19.79 ± 8.35  CG: 25.82 ± 9.41  *p*=0.019  *Days to 100%*  EG: 26.33 ± 10.11  CG: 32.64 ± 9.44  *p*=0.040 |
|  |  | LOS (days) | Median  EG: 43.0  CG: 50.5  *p*=0.028 |
|  | da Rosa Pereira et al. (2020), Brazil | Time to FOF from first oral feed (days) | Median (IQR)  EG: 4 (3–11)  CG: 8 (7–13)  *p*= 0.003 |
|  |  | Milk transfer rate (ml/min) | Median (IQR)  EG: 20 (20–43)  CG: 32 (25–41)  *p*=0.210 |
|  |  | LOS (days) | Median (IQR)  EG: 20 (20–43)  CG: 32 (25–41)  *p*=0.210 |
|  |  | Weight at discharge (g) | Mean ± SD  EG: 2418 ± 461  CG: 2442 ± 519  *p*= 0.940 |
|  | Ghomi et al. (2019), Iran | Time to FOF from initiation of oral feeding (days) | Mean ± SD  EG: 8.07 ± 2.58  CG: 14.33 ± 4.7  *p*< 0.001 |
|  |  | LOS | Mean ± SD  EG: 37.13 ± 11.70  CG: 46.60 ± 11.35  *p*= 0.03 |
|  |  | Weight at discharge (g) | Mean ± SD  EG: 1498.33 ± 128.47  CG: 1546.67 ± 96.84  *p*= 0.25 |
|  | Harding et al. (2014), United Kingdom | Time to FOF from initiation of oral feeding (days) | Average  Group I (NNS pre-tube feeding): 19.7  Group II (NNS on onset of tube feeding): 16.5  Group III (CG): 23.9  *p*= 0.115 |
|  |  | LOS (days) | Mean±SD  Group I (NNS pre-tube feeding): 36.84± 29.96  Group II (NNS on onset of tube feeding): 37.90± 13.94  Group III (CG): 54.40±28.62  *p*=0.022 |
|  | John et al. (2018), India | Time taken for initiation of first breast feed to discharge (days) | Mean ± SD  EG: 11.70 ± 7.53  CG: 9.36 ± 3.44  *p*=0.65 |
|  |  | Behavioral state at start of oral feeding (IBFAT score) | Mean ± SD  *Deep sleep*  EG: 30.33±18.82  CG: 27.85 ± 8.94  *p*=0.88  *Light sleep*  EG: 37.30 ± 10.66  CG: 45.95 ± 10.06  *p*=0.09  *Drowsy*  EG: 42.02 ± 8.94  CG: 42.90 ± 18.30  *p*=0.97  *Quiet alert*  EG: 30.71 ± 8.26  CG: 19.00 ± 5.75  *p*=0.02 |
|  |  | Stimulation required to feed (IBFAT score) | Mean ± SD  *No stimulation*  EG: 60.60 ± 8.99  CG: 58.06 ± 10.80  *p*=0.86  *Elicit rooting*  EG: 36.40 ± 6.78  CG: 40.08 ± 10.37  *p*=0.67  *Unwrap/swaddle*  EG: 32.00  CG: 14.50  *p*=0.32  *Wiggle nipple*  EG: 27.17 ± 6.59  CG: 26.71 ± 5.44  *p*=0.94 |
|  |  | Latching behavior (IBFAT score) | Mean ± SD  *No latch on*  EG: 17.32 ± 7.70  CG: 20.35 ± 70  *p*=0.31  *Partial seal*  EG: 35.71 ± 8.53  CG: 31.70 ± 7.33  *p*=0.26  *Complete seal*  EG: 46.12 ± 5.93  CG: 46.27 ± 11.80  *p*=0.92  *Lick nipple*  EG: 18.00  CG: 7 ± 0.82  *p*=0.14  *Rooting*  EG: 18.59 ± 5.23  CG: 23.35 ± 6.63  *p*=0.06  *No mouth opening*  EG: 16.91 ± 7.16  CG: 21.93 ± 9.12  *p*=0.26  *Partial mouth opening*  EG: 37.57 ± 8.32  CG: 34.55 ± 7.25  *p*=0.20  *Complete mouth opening*  EG: 45.33 ± 5.93  CG: 48.60 ± 11.80  *p*=0.92 |
|  |  | Sucking behavior (IBFAT score) | Mean ± SD  *No sucking*  EG: 20.83 ± 7.82  CG: 18.40 ± 8.40  *p*=0.50  *Mouthing*  EG: 28.21 ± 3.99  CG: 28.22 ± 7.89  *p*=0.65  *Single suck*  EG: 31.38 ± 7.67  CG: 29.27 ± 5.06  *p*=0.40  *Sucking burst*  EG: 24.96 ± 9.66  CG: 28.41 ± 8.92  *p*=0.55  *Time duration of feeding observed (minutes)* EG: 13.70 ± 2.31  CG: 12.66 ± 0.80  *p*=0.32 |
|  |  | LOS (days) | Mean ± SD  EG: 40.1 ± 18.42  CG: 15.3 ± 5.25  *p*=0.32 |
|  |  | Average daily weight gain (kg) | Mean ± SD  EG: 0.63 ± 0.19  CG: 0.48 ± 0.21  *p*=0.10 |
|  | Majoli et al. (2023), Italy | Time to FOF from first oral feed (days) | Mean ± SD  EG: 7.7 ± 5.8  CG: 6.3 ± 3.6  *p*=0.49 |
|  |  | Weight gain (g) | Mean ± SD  EG: 1.879 ± 351  CG: 1.783 ± 270  *p*=0.40 |
|  |  | LOS (days) | Mean ± SD  EG: 41.3 ± 12.6  CG: 46.2 ± 14.0  *p*=0.26 |
|  | Skaaning et. al (2020), Denmark | Strength of suction (intra-oral peak vacuum at corrected age of 6 weeks; mbar) | Mean ± SD  EG: 374 ± 80  CG: 395 ± 93  *p*=0.08 |
|  | Song et al. (2019), USA | Time to FOF from initiation of oral feeding (days) | Mean ± SD  EG: 22.9 ± 10.5  CG: 27.0 ± 14.8  *p*=0.04 |
|  |  | LOS (days) | Mean ± SD  EG: 59.8 ± 20.2  CG: 65.8 ± 19.8  *p*=0.04 |
|  | Thakkar et al. (2018), India | Time to FOF from initiation of oral feeding (days) | Mean ± SD  EG: 8.58 ± 1.05  CG: 13.16 ± 0.71  *p*=0.001 |
|  |  | Milk volume intake (mg/kg/feed) | Mean ± SD  *Initiation of oral feeding*  EG: 3.15 ± 0.64 CG: 3.08 ± 0.52  *p*=0.55  *5th day of intervention*  EG: 6.59 ± 0.77 CG: 6.1 ± 0.58 *p*<0.001  *FOF*  EG:10.37 ± 0.71 CG: 9.81 ± 0.48 *p*<0.001 |
|  |  | Milk transfer rate (ml/min) | Mean ± SD  *Initiation of oral feeding*  EG: 0.95 ± 0.41 CG: 0.9 ± 0.24  *p*=0.46  *5th day of intervention*  EG: 2.19 ± 0.66 CG: 1.77 ± 0.5 *p*=<0.001  *FOF*  EG: 2.98 ± 0.73 CG: 2.58 ± 0.52 *p*<0.001 |
|  |  | Weight gain (g) | Mean ± SD  EG: 20.33 ± 2.67  CG: 15.60 ± 2.66  *p*<0.001 |
|  |  | LOS (days) | Mean ± SD  EG: 22.12 ± 1.88  CG: 24.88 ± 2.09  *p*<0.001 |
| Swallowing Exercise | Heo et al. (2022), South Korea | Time to independent oral feeding from start of oral feeding (days) | Mean ± SD  Group I (CG): 21.1 ± 15.3  Group II (DST): 17.2 ± 9.9  Group III (DST + OMS): 14.8 ± 9.4  *p*=0.02  Post hoc analysis *p*-values  Group I vs Group II: 0.24  Group II vs Group III: 0.45  Group I vs Group III: 0.02 |
|  |  | Feeding proficiency (% volume taken in first 5 minutes of oral feeding) | Mean difference  *Proficiency at start of oral feeding*  Group III vs Group I: 7.8%, *p*=0.02  Group III vs Group II: 6.6%, *p*=0.04  *Proficiency at 100% oral feeds of daily intake*  Group II vs Group I: 7.0%, *p*=0.04  Group III and Group I: 5.19%, *p*=0.12 |
|  |  | LOS (days) | Mean ± SD  Group I (CG): 60.8 ± 24.0  Group II (DST): 57.9 ± 23.0  Group III (DST + OMS): 53.2 ± 24.1  *p*=0.12 |
|  |  | Change of body weight z-score from birth to discharge | Mean ± SD  Group I (CG): -1.51 ± 1.98  Group II (DST): -1.08 ± 0.57  Group III (DST + OMS): -1.22 ± 0.71  *p*=0.16 |
|  | Ostadi et al. (2021), Iran | Time to FOF from start of oral feeding (days) | Mean ± SD  Group I (NNS + NNS): 8.6 ± 7.1  Group II (SE + NNS): 7.1 ± 7.2  Group III (CG):12.4 ± 6.2  *p*=0.14 |
|  |  | PMA at FOF (wk) | Mean ± SD  Group I (NNS + NNS):32.9 ± 0.9  Group II (SE + NNS): 33.7 ± 1.7  Group III (CG):33.9 ± 1.2  *p*=0.13 |
|  |  | Discharged without tube feeding (%) | Group I: 53.9 vs Group II: 78.6 *p*=0.17  Group II: 78.6 vs Group III 30.8 *p*=0.013  Group I: 53.9 vs Group III 30.8 *p*=0.23 |
|  |  | Feeding readiness (POFRAS score) | Post-hoc comparisons of mean POFRAS  Group I vs Group II *p*=0.98  Group II vs Group III *p*=0.013  Group I vs Group III *p*=0.025 |
|  |  | PMA at discharge (wk) | Mean ± SD  Group I (NNS + NNS):33.7 ± 2.7  Group II (SE + NNS): 34.5 ± 2.0  Group III (CG):33.4 ± 1.7  *p*=0.45 |
| Sensory-based | Alidad et al. (2021), Iran | Time to FOF from initiation of intervention (days) | Mean ± SD  EG: 8.04 ± 2.8  CG: 12.27 ± 6.47  *p*=0.007 |
|  |  | POFRAS score | Mean POFRAS score before treatment (T0), after 7th session (T1), after 14th session (T2), and 7 days post-intervention (T3)  Mean ± SD  EG  T0: 19.9 ± 3.81  T1: 26.81 ± 4.07  T2: 31.9 ± 1.9  T3: 34.86 ± 0.8  CG  T0: 18.8 ± 4.2  T1: 24.4 ± 5  T2: 28.37 ± 4.5  T3: 33.2 ± 3.8  Group effect: *p*=0.03  Time x Group interaction: *p*=0.45 |
|  |  | Milk volume intake (ml) | Milk volume intake at T0, T1, T2, and T3  Mean ± SD  EG  T0: 1.43 ± 1.05  T1: 10.22 ± 7.48  T2: 20.59 ± 10.2  T3: 30.5 ± 12.1  CG  T0: 1.03 ± 0.87  T1: 4.59 ± 3.08  T2: 9 ± 4.2  T3: 14.5 ± 5.8  Group effect: *p*<0.0001  Time x Group interaction: *p*=0.61 |
|  |  | LOS (days) | Mean ± SD  EG: 20.27 ± 10.9  CG: 21.7 ± 15.4  *p*=0.72 |
|  |  | Weight gain (g) | Weight gain at T0, T1, T2, and T3  Mean ± SD  EG  T0: 2026.81 ± 627.1  T1: 2080.9 ± 638.1  T2: 2191.8 ± 628.3  T3: 2318.1 ± 629.1  CG  T0: 2158.1 ± 694.4  T1: 2147.9 ± 690.8  T2: 2208.1 ± 710.5  T3: 2291.5 ± 750.7  Group effect: *p*=0.67  Time x Group interaction: *p*=0.07 |
|  | Hernández Gutiérrez et al. (2022), Spain | Time to independent oral feeding (days) | Mean ± SD  *Independent oral feeding (from start of intervention)*  EG: 13.8 ± 9.5  CG: 19.1 ± 10.4  *p*=0.09  *Full independent oral feeding (from birth)*  EG: 24.9 ± 10.1  CG: 34.1 ± 15.6  *p*=0.02 |
|  |  | LOS (days) | Mean ± SD  EG: 39.0 ± 15.3  CG: 45.3 ± 17.6  *p*=0.21 |
|  | Shokri et al. (2023), Iran | Time to FOF (reaching 8 oral feeds per day; days) | Mean ± SD  EG: 31.54 ± 8.6  CG: 39.19 ± 13.3  *p*=0.018 |
|  |  | Day 10 POFRAS score | Mean ± SD  EG: 28.65 ± 3.0  CG: 20.96 ± 3.3  *p*=0.001 |
|  |  | Milk volume intake (ml) | Mean ± SD  EG: 215.38 ± 38.3  CG: 155.69 ± 48.1  *p*=0.001 |
|  |  | LOS (days) | Mean ± SD  EG: 44.96 ± 16.2  CG: 50.65 ± 17.1  *p*=0.224 |
|  |  | Weight gain (g) | Mean ± SD  EG: 1388.54 ± 171.3  CG: 1350.96 ± 243.2  *p*=0.522 |

FOF = full oral feeding, EG = experimental group, CG = control group, SD = standard deviation, LOS = length of hospital stay, IQR = interquartile range, IBFAT = Infant Breastfeeding Assessment Tool, DST = direct swallowing training, OMS = oral motor stimulation, NNS = non-nutritive sucking, SE = swallowing exercise, PMA = postmenstrual age, POFRAS = Preterm Oral Feeding Readiness Scale
